# Cigarette use, secondhand smoke exposure and age-related infertility among US women: A cross-sectional NHANES study

**DOI:** 10.18332/tpc/217327

**Published:** 2026-07-09

**Authors:** Yuyan Li, Ying Zhou, Bingxue Wu, Yan Zhang, Yishi Jiang, Junqing Wu, Yan Che

**Affiliations:** 1 NHC Key Lab of Reproduction Regulation, Shanghai Engineering Research Center of Reproductive Health Drug and Devices, Shanghai Institute for Biomedical and Pharmaceutical Technologies, Shanghai, China; 2 Department of Research and Development, Shanghai Proton and Heavy Ion Center, Shanghai Key Laboratory of Radiation Oncology, Shanghai Engineering Research Center of Proton and Heavy Ion Radiation Therapy, Shanghai, China

**Keywords:** cigarette smoking, secondhand smoke, age-related infertility, serum cotinine

## Abstract

**INTRODUCTION:**

While tobacco exposure is linked to infertility, age-specific risks across different exposure groups remain poorly characterized. This study aimed to examine the associations of active cigarette smoking and secondhand smoke (SHS) exposure with female infertility and to assess potential effect modification by age.

**METHODS:**

This is a cross-sectional secondary analysis of publicly available data from the National Health and Nutrition Examination Survey (NHANES, 2013–2020 cycles). The primary exposures were self-reported active cigarette smoking, SHS exposure (assessed via questionnaire and serum cotinine). The outcome was self-reported infertility. Survey-weighted restricted cubic spline (RCS) and logistic regression models were used to assess the respective associations.

**RESULTS:**

This study comprised 4982 women aged 18–49 years. The overall prevalence of self-reported infertility was 12.0% (95% CI: 10.7–13.2), with the highest prevalence observed at approximately 40 years. The association between tobacco smoke exposure and infertility showed a significant negative interaction with age, observed for both self-reported smoking status and serum cotinine levels, and was most pronounced among women aged 18–40 years. In weighted multivariable logistic regression models fitted to the full sample, at age of 18 years, former smokers had significantly higher odds of infertility than never smokers (OR=2.78; 95% CI: 1.30–5.96). Also at this age, when using serum cotinine <0.05 ng/mL as the reference (unexposed), higher odds were observed both in the SHS-exposed group (0.05–10 ng/mL; OR=2.59; 95% CI: 1.25–5.38) and the active-smoking-exposed group (>10 ng/mL; OR=3.79; 95% CI: 1.86–7.69).

**CONCLUSIONS:**

Among women aged 18–40 years, cigarette smoking and greater tobacco smoke exposure, indicated by serum cotinine, were associated with infertility. Public health efforts should prioritize smoking prevention, cessation, and reduced secondhand smoke exposure to support female reproductive health.

## INTRODUCTION

Declining female fertility and infertility have become global health concerns^1^. Infertility is defined as the failure to achieve pregnancy after 12 months of regular unprotected intercourse^2,3^, with an estimated prevalence in reproductive-aged women of one in seven couples in high-income countries and one in four couples in developing regions as of 2010^3^. In the United States, the proportion of infertile married women aged 15–44 years increased from 6.7% (2011–2015) to 8.7% (2015–2019), which may be related to advanced maternal age^4^. The success of human reproduction is highly dependent on age. Reproductive aging is a natural process and female aging is one of the most important factors that impacts reproduction, characterized by age-related follicle depletion and a reduction in oocyte quality, which eventually leads to reproductive senescence in females^5^. Advanced age is one of the critical risk factors for female-related infertility^6^.

Although reproductive aging is inevitable, modifiable lifestyle factors may influence fertility and could potentially mitigate infertility risk. Substantial attention has focused on preventable exposures, including unhealthy behaviors such as cigarette smoking, in relation to female fertility^7^. Smoking is of particular concern given its well-established links to a wide range of diseases. In the United States, 20.8% of adults in 2019 and 19.0% in 2020 reported any tobacco use. Tobacco use was more common among adults aged 25–44 years (25.3% in 2019 and 22.9% in 2020), and cigarettes remained the most frequently used tobacco product^8,9^.

Accumulating epidemiological evidence has established an association between tobacco exposure and female infertility. Previous studies have consistently demonstrated elevated infertility risks among active smokers, with large cohort studies further suggesting a dose-response relationship and adverse effects of secondhand smoke (SHS)^10-12^. Analyses of the National Health and Nutrition Examination Survey (NHANES) have strengthened these findings, linking both self-reported smoking status and biomarker-quantified exposure (e.g. serum cotinine) to higher infertility prevalence^13,14^. Lei et al.^15^ revealed that higher oxidative balance score (serum cotinine as one of the pro-oxidant components) was negatively associated with female infertility. In addition, smoking is frequently incorporated as a key covariate and effect-modifying factor in infertility research^16-18^.

Nevertheless, the overall impact of tobacco exposure on fertility and reproduction remains incompletely understood^19^, and age-specific patterns of infertility across smoking exposure strata have not been fully characterized. To address these gaps, an analysis was conducted using NHANES 2013–2020 data to further evaluate the associations of active cigarette smoking and SHS exposure with female infertility, thereby providing additional evidence to inform prevention and public health efforts aimed at improving female reproductive health.

## METHODS

### Study design and data sources

This study conducted a secondary analysis of data from NHANES, a cross-sectional survey, to evaluate the association between cigarette smoking and female infertility. NHANES is a continuous, population-based program administered by the Centers for Disease Control and Prevention (CDC)^13,20^. It employs a complex, four-stage, stratified sampling design to generate nationally representative estimates. Participants complete an in-home interview and subsequently attend a Mobile Examination Center, where standardized physical examinations are performed and biological specimens are collected^18,21^.

### Study participants

**Figure 1:**
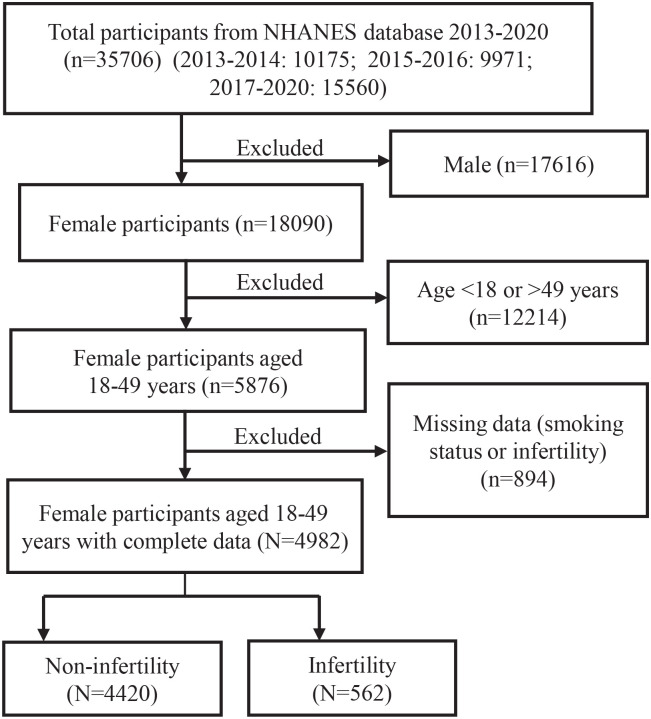
Flow chart of the study participants, cross-sectional analysis of NHANES 2013–2020, United States (N=4982)

Participants were drawn from three NHANES cycles that included the female infertility questionnaire: two 2-year cycles (2013–2014 and 2015–2016) and the combined 2017–March 2020 cycle. The analytic sample was restricted to women of reproductive age (18–49 years). Participants with missing information on smoking status or infertility (n=894) were excluded (Figure 1). NHANES protocols were approved by the relevant ethics review board, and written informed consent was obtained from all participants prior to study participation.

### Study indicators

*Outcome indicator*

The outcome indicator was self-reported ‘whether or not having infertility’ (RHQ074: ‘Tried for a year to become pregnant?’). Participants answering ‘yes’ were classified into the infertility group, and those answering ‘no’ into the non-infertility group.

*Independent factors*

The main research factors were active-smoking and passive-smoking (serum cotinine as an indicator of tobacco exposure).

Cigarette smoking in this study was defined as ‘smoked at least 100 cigarettes in life’. The grouping of current smoking status was generated based on the variables SMQ020 (Smoked at least 100 cigarettes in life, ‘yes’ or ‘no’) and SMQ040 (‘Do you now smoke cigarettes?’, ‘yes’ or ‘no’) as ‘never smoked’, ‘ever smoked’, and ‘current smoker’, respectively.

The indictor of ‘whether having passive smoking (yes or no)’ was calculated based on the NHANES Secondhand Smoke Exposure Survey. Those who are not sure whether they have passive smoking or not, are defined as the ‘unclear’ group to improve statistical efficiency.

Serum cotinine concentration (LBXCOT; ng/mL), a biomarker of recent tobacco smoke exposure, was measured in serum samples collected at the Mobile Examination Center (MEC) using isotope-dilution high-performance liquid chromatography coupled with atmospheric pressure chemical ionization tandem mass spectrometry (ID-HPLC-APCI-MS/MS). Results below the 0.015 ng/mL LLOD are flagged (LBDCOTLC=1) and imputed as LLOD/√2. QA/QC follows NHANES LPM and CLIA rules, with blind split samples.

*Potential covariates*

The covariates of this study included age (18–49 years) (treated as continuous variable, and grouped by age at 40), race/ethnicity (categorized as standard NHANES groups, e.g. Mexican American, Other Hispanic, Non-Hispanic White, Non-Hispanic Black, Other), education level (categorized as lower than 9th, lower than 11th, high school graduate, some college/AA degree, college graduate or higher), ratio of family income to poverty (PIR) (treated as continuous variable), body mass index (BMI, kg/m^2^, treated as continuous variable), current alcohol drinking status (categorized as ‘No’, ’Yes’ two groups), whether having hypertension (categorized as ‘No’, ’Yes’ two groups) and whether having diabetes mellitus (categorized as ‘No’, ’Yes’ two groups).

### Statistical analysis

Data analyses were conducted using R4.4 software. A two-tailed p<0.05 was considered statistically significant. Data from the three cycles (2013–2014, 2015–2016, 2017–March 2020) was combined and the weights were recalculated. All analyses accounted for the complex survey design of NHANES.

Baseline characteristics were compared between the infertility and control groups, including age, race/ethnicity, education level, marital status, BMI, PIR, alcohol drinking status, and presence of hypertension or diabetes (Table 1). For continuous variables (age and BMI), weighted means and standard deviations (SDs) were calculated, and group differences were assessed using weighted linear regression. Because PIR was right-skewed, weighted medians and percentiles were reported and compared using a weighted rank-sum test; PIR was then log-transformed for subsequent analyses (log PIR = ln [PIR + 1]) to reduce skewness and avoid negative values. For categorical variables, weighted chi-squared tests were applied.

**Table 1:** Comparison of basic characteristics* of women between two groups, cross-sectional analysis of NHANES 2013–2020, United States (N=4982)

*Characteristics*	*Non-infertility* *group* *%*	*Infertility* *group* *%*	*p*
**Age (**years), mean (SD)	33.10 (9.32)	36.78 (7.81)	<0.001
**BMI** (kg/m²), mean (SD)	29.01 (8.06)	32.12 (9.11)	<0.001
**PIR,** median (IQR)	2.63 (1.23–4.62)	2.98 (1.55–4.78)	0.018
**Race/ethnicity**			
Mexican American	11.83	9.65	0.173
Non-Hispanic Black	13.29	12.62	
Non-Hispanic White	56.70	61.73	
Other Hispanic	7.90	7.61	
Other Race	10.28	8.39	
**Education level**			
Lower than 9th	3.30	1.94	0.362
Lower than 11th	8.72	8.04	
High school graduate	20.17	18.55	
Some college/AA degree	33.92	36.15	
College graduate or higher	33.89	35.32	
**Alcohol drinking**			
No	20.97	21.06	0.969
Yes	79.03	78.94	
**Hypertension**			
No	86.11	78.13	0.002
Yes	13.89	21.87	
**Diabetes**			
No	96.53	90.84	<0.001
Yes	3.47	9.16	

* Sample weighted. IQR: interquartile range.

**Figure 2:**
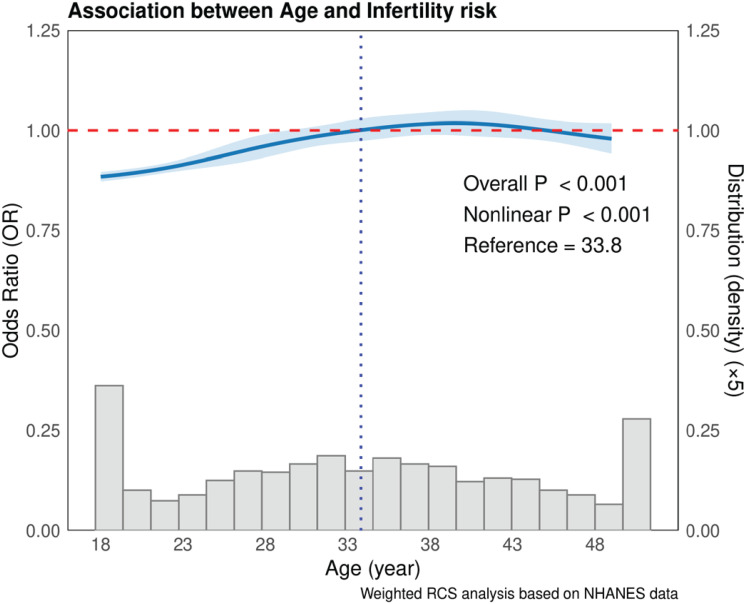
Weighted RCS analysis between age and infertility, cross-sectional analysis of NHANES 2013–2020, United States (N=4982)

**Figure 3:**
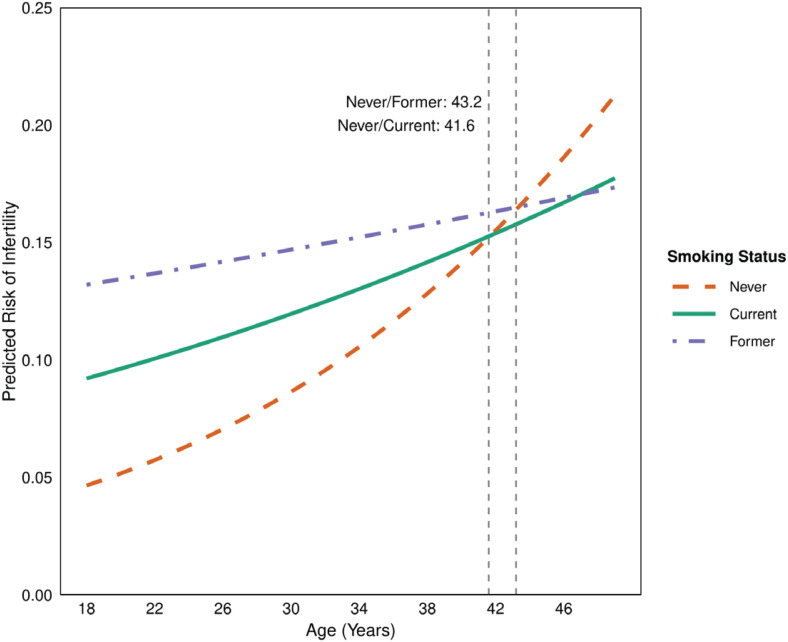
The interaction between active smoking, age, and infertility, cross-sectional analysis of NHANES 2013–2020 , United States (N=4982)

Survey-weighted restricted cubic spline (RCS) analyses were conducted for age, BMI and PIR (log) to examine their associations with infertility [Figure 2 for result of age, no figure listed for BMI and PIR (log)]. Weighted chi-squared test was used to compare infertility prevalence across groups defined by active and passive smoking status (Table 2). The interaction analysis was conducted to evaluate potential modification between age and smoking status (Figure 3). For serum cotinine, the geometric means (GMs) and 95% confidence intervals (CIs)^22^ were calculated using weighted method across different smoking status groups, and weighted linear regression was used for comparisons (no table listed). According to the serum cotinine ranges 0.05–10 ng/mL^23^, it was classified into three groups (non-exposure group:<0.05 ng/mL, SHS-exposed group: 0.05–10 ng/mL, active smoking-exposed group: >10 ng/mL), and weighted logistic regression was subsequently employed to further analyze the association with age-infertility patterns. Weighted multivariable logistic regressions and some subgroups analyses according to age (cutoff: 40 years) were performed to explore the associations between smoking exposure and infertility (Tables 3 and 4, and Supplementary file Tables S1 and S2).

**Table 2:** Comparison of infertility prevalence* among different smoking groups, cross-sectional analysis of NHANES 2013–2020, United States (N=4982)

*Characteristics*	*Total* *n*	*Non-* *infertility* *group* *%*	*Infertility* *group* *%*	*p*
**Active smoking**				
Never	3591	86.70	13.30	0.009
Former	554	84.35	15.65	
Current	837	89.16	10.84	
**Passive smoking**				
No	140	93.39	6.61	0.160
Yes	1447	86.80	13.20	
Unclear	3395	88.19	11.81	

* Sample weighted.

**Table 3:** Weighted logistic regression of the association between active smoking, age, and infertility, cross-sectional analysis of NHANES 2013–2020, United States (N=4982)

*Variables*	*Comparison group*	*OR*	*95% CI*	*p*
**Bivariable Model 1a**				
**Age** (baseline=18 years)	Continuous	1.06	1.04–1.07	<0.001
**Smoking** (ref: Never)	Current	2.08	1.06–4.08	0.034
	Former	3.12	1.53–6.35	0.024
**Interaction item**	Age × Current	0.97	0.94–0.999	0.046
	Age × Former	0.96	0.92–0.99	0.001
**Multivariable Model 1b**				
**Age** (baseline=18 years)	Continuous	1.05	1.03–1.07	<0.001
**Smoking** (ref: Never)	Current	1.83	0.87–3.83	0.096
	Former	2.78	1.30–5.96	<0.001
**Interaction item**	Age × Current	0.97	0.94–1.01	0.122
	Age × Former	0.96	0.92–0.99	0.015

**Table 4:** Weighted logistic regression of the association between serum cotinine, age, and infertility, cross-sectional analysis of NHANES 2013–2020, United States (N=4702)

*Variables*	*Comparison group*	*OR*	*95% CI*	*p*
**Bivariable Model 2a**				
**Age** (baseline=18 years)	Continuous	1.06	1.05–1.08	<0.001
**Cotinine group** (Ref: <0.05 ng/mL)	0.5–10	2.10	1.13–3.89	<0.001
	>10	3.07	1.68–5.60	<0.001
**Interaction item**	Age × 0.5–10	0.98	0.94–1.01	0.244
	Age × >10	0.95	0.93–0.98	<0.001
**Multivariable Model 2b**				
**Age** (baseline=18 years)	Continuous	1.06	1.04–1.08	<0.001
**Cotinine group** (Ref: <0.05 ng/mL)	0.5–10	2.59	1.25–5.38	<0.001
	>10	3.79	1.86–7.69	<0.001
**Interaction item**	Age × 0.5–10	0.97	0.93–1.01	0.112
	Age × >10	0.95	0.92–0.96	<0.001

Multivariable analysis adjusted for race/ethnicity, education level, BMI, PIR (log), alcohol drinking, hypertension, and diabetes.

## RESULTS

### Basic characteristics

A total of 4982 women aged 18–49 years were included in the final analysis. Survey-weighted linear regression indicated that women reporting infertility were significantly older than those in the non-infertility group (36.78 ± 7.81 vs 33.10 ± 9.32 years; p<0.001). BMI and PIR also differed significantly between groups (both p<0.05). In addition, the prevalence of hypertension (p=0.002) and diabetes (p<0.001) was significantly higher in the infertility group. In contrast, no significant between-group differences were observed for race/ethnicity, education level, or alcohol drinking status (Table 1). Significant linear associations between infertility and BMI as well as log-transformed PIR were also observed (no figure listed).

### Age and infertility

In this study, the overall infertility prevalence was 12.0% (95% CI: 10.7–13.2). A weighted RCS analysis showed that the reported infertility rate was highest at 39.61 years (approximately 40 years) at 16.50% (95% CI: 13.44–19.56) (Figure 2).

### Cigarette smoking and infertility

A statistically significant difference in infertility prevalence was observed across categories of active smoking status (13.30% among never smokers and 15.65% among former smokers). By contrast, infertility prevalence did not differ significantly across passive smoking exposure categories (13.20% in the passive smoking group, 11.81% in the ‘unclear’ group, and 6.60% in the non-exposure group; p=0.160).

In the survey-weighted bivariable logistic model (Table 3, Model 1a), the interaction terms between age and active smoking status were statistically significant (p<0.05). Among never smokers, each one-year increase in age was associated with a 6% increase in the odds of infertility (OR=1.06; 95% CI: 1.04–1.07). At age 18 years, both former smokers (OR=3.12; 95% CI: 1.53–6.35) and current smokers (OR=2.08; 95% CI: 1.06–4.08) had significantly higher odds of infertility compared with never smokers. As shown in Figure 3, the predicted infertility probability at age 18 years was highest among former smokers, and this group demonstrated the smallest age-related increase. These patterns were further evaluated using a weighted multivariable logistic regression model (Table 3, Model 1b) adjusting for demographic and clinical covariates. Overall, results from the adjusted model were consistent with the bivariable model (Model 1a), except that the association for current smokers was attenuated and no longer statistically significant. In contrast, no significant interaction between age and passive smoking status was detected, and the multivariable model (Model 1b) did not identify significant differences in infertility risk across passive smoking exposure categories (results not shown).

Multivariable analysis adjusted for race/ethnicity, education level, BMI, PIR (log), alcohol drinker, hypertension, diabetes, and passive smoking.

Stratified analyses indicated that the interaction between active smoking status and age was primarily present among women 18–40 years, and this pattern was observed in both the bivariable and multivariable models (Supplementary file Table S1, Models a1 and a2). In the multivariable model, at age 18 years, compared with never smokers, current smokers (adjusted odds ratio, AOR=3.10; 95% CI: 1.14–8.39) and former smokers (AOR=4.83; 95% CI: 1.78–13.09) had significantly higher odds of infertility (Supplementary file Table S1 Model a2). In contrast, among women aged ≥40 years, no statistically significant associations between smoking status and infertility were detected (Supplementary file Table S1 Model b1 and b2).

### Serum cotinine and infertility

Survey-weighted geometric means (GMs; 95% CIs) of serum cotinine were 101.23 ng/mL (83.95–122.06) among current smokers, 0.17 ng/mL (0.11–0.26) among former smokers, and 0.04 ng/mL (0.03–0.043) among never smokers (results not tabulated). Serum cotinine levels were significantly higher among current and former smokers than among never smokers (p<0.001). Weighted logistic regression models identified a negative interaction between the active smoking-exposed group (>10 ng/mL) and age with respect to infertility, both in the bivariable model (Table 4, Model 2a) and in the multivariable model adjusted for race/ethnicity, education level, BMI, log-transformed PIR, alcohol consumption, hypertension, and diabetes (Model 2b). Using cotinine <0.05 ng/mL as the reference (n=2568), at age 18 years, the odds of infertility were higher in the SHS-exposed group (n=1127; OR=2.59; 95% CI: 1.25–5.38) and in the active smoking–exposed group (n=1007; OR=3.79; 95% CI: 1.86–7.69). Stratified analyses further indicated that the interaction between cotinine groups and age was primarily evident among women 18–40 years, with both exposure categories demonstrating negative interactions with age (Supplementary file Table S2, Models c1 and c2). In contrast, among women aged ≥40 years, no statistically significant differences in infertility were observed across cotinine exposure categories (Supplementary file Table S2, Models d1 and d2).

## DISCUSSION

Our analysis showed that the likelihood of female infertility increased with age, peaking at around 40, which is consistent with prior evidence that female fecundity declines markedly in the late 30s to early 40s^24^. Higher infertility prevalence was also observed among both former and current smokers, and this association was most evident among women aged 18–40 years. A large cohort study has reported that lifetime tobacco exposure – including active smoking and secondhand smoke – was statistically associated with outcomes such as a history of infertility^25^. However, the literature is not fully consistent regarding whether ever smoking (vs never smoking) remains independently associated with infertility after rigorous control for confounding. For example, a combined multivariable regression and Mendelian randomization analysis did not find a clear association between ever smoking and infertility^12^. Prospective preconception cohorts have reported heterogeneous results, with less consistent associations for passive smoke exposure and, in some settings, limited or null differences in fecundability for certain exposure metrics after adjustment^26^. In the present cross-sectional analysis, the absence of a statistically significant association for passive smoking as assessed by questionnaire may reflect limited statistical power in the exposed subgroup, as well as exposure misclassification, including the presence of a sizable ‘unclear’ category. Moreover, self-reported passive exposure may be particularly prone to measurement error because SHS intensity is intermittent and context-dependent, which could attenuate associations toward the null.

Biomarker-defined tobacco exposure, assessed using serum or urinary cotinine, has been linked to higher infertility prevalence in prior studies^19^. In this cross-sectional analysis, higher serum cotinine was also associated with infertility, and NHANES-based work suggests the relationship may be non-linear and sensitive to cut points and analytic approaches^14^. In contrast, an assisted reproduction study reported no association between urinary cotinine above the median and failed fertilization, implantation failure, or spontaneous abortion, underscoring heterogeneity across exposure windows, populations, and outcome definitions^27^. Because cotinine reflects recent exposure, a single measurement may not capture long-term or intermittent secondhand smoke exposure and could attenuate associations; reverse causation is also possible if women with fertility concerns modify smoking behavior. Nevertheless, elevated odds were observed in both SHS- and active smoking–exposed cotinine groups, supporting the public health relevance of reducing secondhand smoke exposure. Finally, given evidence that many US non-smokers underreport nicotine exposure^21^, and our results of self-reported smoking status and serum cotinine suggests that self-report of current smoking may underestimate true smoking prevalence. These findings underscore the need for enhanced regulatory monitoring and efforts to lower environmental nicotine levels.

In this analysis, no statistically significant associations between cigarette smoking, serum cotinine levels, and female infertility were observed among women aged ≥40 years, a group in which infertility prevalence is already elevated due to reproductive aging. Similar age-specific attenuation has been reported previously; for example, He et al.^13^ found that the fully adjusted OR comparing current smokers with never smokers was not statistically significant in women aged 39–45 years. This may be related to the predominance of age effects, insufficient sample size/statistical power, etc. Associations between smoking-related exposures and infertility can vary by exposure indicator and analytic approach, and the underlying pathways are unlikely to be linear or uniform across populations^12,26^. Overall, tobacco exposure has been frequently associated with higher infertility prevalence, whereas age appears to be the dominant correlate of infertility in older reproductive-age women^10,12,19^. Age-related infertility has been recognized as one of ten key research priorities in infertility studies^28^. These findings underscore the importance of supporting reproductive health across the life course, including promoting timely childbearing within the optimal reproductive window and reinforcing smoking prevention and cessation.

An interaction between smoking status and age with respect to infertility was observed and should be interpreted cautiously. This pattern may reflect: 1) reverse causation (e.g. quitting in response to fertility concerns, inflating risk among ‘former’ smokers)^12^, 2) residual confounding and differential health behaviors by age^10,19^, and/or 3) a ceiling effect whereby infertility becomes common at older ages, reducing statistical contrast across exposure groups^29^. Despite these considerations, the broader literature supports an adverse relationship between tobacco exposure and female infertility^10^, and mechanistic and clinical literature supports the plausibility that tobacco exposure can accelerate reproductive aging through effects on ovarian reserve and follicular function, although findings across clinical populations are not uniform^19,30^. Overall, these mixed findings across different study designs underscore the necessity for more detailed and repeated measures of smoking history, including pack-years, intensity, and time since cessation, as well as of SHS exposure in future longitudinal research. Additionally, evidence suggests that some smoking-related impairments in fertility may diminish after cessation^19,31,32^. Accordingly, smoking cessation remains an actionable strategy to support reproductive health^13,33^. Therefore, it remains a clear clinical and public health priority to reduce both active smoking and secondhand smoke exposure throughout the reproductive life course.

### Limitations

Several limitations should be acknowledged. First, smoking status and infertility were both self-reported, which may be influenced by social desirability bias and recall error. Underreporting of smoking or inaccuracies in fertility history could result in exposure and outcome misclassification, potentially attenuating or distorting observed associations. Second, misclassification bias may also arise from the use of broad or heterogeneous exposure categories, such as ‘former smoking’ or passive exposure, which can include individuals with varied exposure intensity, duration, and timing, thereby diluting between-group contrasts. Third, as with all observational analyses, residual confounding cannot be ruled out. Although we adjusted for key sociodemographic factors, unmeasured or imprecisely measured confounders may partially explain the observed associations. Finally, participants with missing data were excluded, which may introduce selection bias if data were not missing completely at random, potentially limiting generalizability and biasing the estimates.

### Future research

Future studies should adopt prospective preconception designs featuring repeated and objective exposure assessments – such as serial cotinine measurements and complementary biomarkers reflecting long-term exposure – along with detailed characterization of smoking patterns (including intensity, duration, cessation timing, and sources of secondhand smoke) and standardized definitions of infertility (e.g. time-to-pregnancy and clinically confirmed diagnoses).

## CONCLUSIONS

Female infertility was strongly associated with age, with the highest prevalence observed in the late reproductive years (approximately age 40 years). Tobacco smoke exposure, both active smoking and secondhand exposure indexed by serum cotinine, was associated with infertility, particularly among women aged 18–40 years. These findings support public health efforts to prevent smoking, promote cessation, and reduce secondhand smoke exposure to protect female reproductive health and potentially lower infertility risk.

## Supplementary Material



## Data Availability

The data supporting this research are available from the following source: https://www.cdc.gov/nchs/nhanes/
