# Supplementary material for: Cigarette use, secondhand smoke exposure and age-related infertility among US women: A cross-sectional NHANES study
Source: Tob Prev Cessat. 2026 Jul 9;12:10.18332/tpc/217327. doi: 10.18332/tpc/217327 (PMC13352491; doi:10.18332/tpc/217327)
Supplement: Supplementary file 1 [file TPC-12-34-s1.pdf]

 DOI:10.18332/tpc/217327

Supplementary materials-analysis results

Table S1 Weighted logistic regression analysis of the association between active smoking, age, and infertility, cross-sectional analysis of NHANES 2013-2020 (United States)

Variables	Comparison group	Reference group	**Model a (age<40)** (n=3429)			**Model b (age≥40)** (n=1553)		
			OR	95%CI	P value	OR	95%CI	P value
Bivariable models				Model a1			Model b1	
Age (baseline=18)	Continuous		1.12	1.09-1.14	<0.001	0.86	0.89-1.04	0.283
Smoker Current	Current	Never	3.64	1.51-8.78	0.005	1.17	0.77-1.77	0.460
	Former		5.77	2.31-14.41	<0.001	1.01	0.68-1.51	0.950
Interaction item	Age: Current		0.91	0.86-0.97	0.002	—	—	—
	Age: Former		0.91	0.85-0.97	0.004	—	—	—
Multi-variable models				Model a2			Model b2	
Age (baseline=18)	Continuous		1.10	1.07-1.14	<0.001	0.93	0.85-1.02	0.124
Smoker Current	Current	Never	3.10	1.14-8.39	0.005	1.15	0.73-1.81	0.529
	Former		4.83	1.78-13.09	<0.001	0.80	0.52-1.24	0.314
Interaction item	Age: Current		0.92	0.86-0.98	0.010	—	—	—
	Age: Former		0.91	0.85-0.98	0.012	—	—	—

Note: OR: odds ratio; CI: Confidence Interval;

Multi-variable analysis adjusted for race/ ethnicity, education, BMI, PIR (log), alcohol drinker, hypertension, diabetes and passive smoking.

SUPPLEMENTARY FILE DISCLAIMER

The content has been provided by the author(s) and has not been reviewed, verified, or endorsed by European Publishing. It may not have undergone peer review. The views, opinions, and recommendations expressed are solely those of the author(s) and do not necessarily reflect the position of European Publishing. European Publishing accepts no responsibility or liability for any consequences arising from the use of, or reliance on, this content.

 DOI:10.18332/tpc/217327

Table S2 Weighted logistic regression analysis of the association between serum cotinine, age, and infertility, cross-sectional analysis of NHANES 2013-2020 (United States)

Variables	Comparison group	Refere-nce	**Model c (age<40)** (n=3208)			**Model d (age≥40)** (n=1494)		
			OR	95%CI	P value	OR	95%CI	P value
Bivariable models				Model c1			Model d1	
Age (baseline=18)	Continuous		1.14	1.10-1.18	<0.001	0.96	0.88-1.04	0.277
Cotinine group(ng/mL)	0.5-10	<0.05	3.65	1.56-8.54	0.004	1.14	0.59-2.22	0.691
	>10		6.74	2.69-16.89	<0.001	1.03	0.68-1.56	0.876
Interaction item	Age: 0.5-10		0.95	0.90-1.00	0.049	—	—	—
	Age: >10		0.89	0.84-0.95	<0.001	—	—	—
Multi-variable models				Model c2			Model d2	
Age (baseline=18)	Continuous		1.13	1.08-1.18	<0.001	0.93	0.85-1.02	0.136
Cotinine group(ng/mL)	0.5-10	<0.05	4.27	1.55-11.76	0.006	1.15	0.57-2.31	0.691
	>10		7.66	2.67-22.01	<0.001	1.18	0.75-1.85	0.460
Interaction item	Age: 0.5-10		0.93	0.88-1.10	0.039	—	—	—
	Age: >10		0.89	0.83-0.95	<0.001	—	—	—

Note: OR: odds ratio; CI: Confidence Interval;

Multi-variable analysis adjusted for race/ ethnicity, education, BMI, PIR (log), alcohol drinker, hypertension and diabetes.

© **2026 Li Y. et al.**

SUPPLEMENTARY FILE DISCLAIMER

The content has been provided by the author(s) and has not been reviewed, verified, or endorsed by European Publishing. It may not have undergone peer review. The views, opinions, and recommendations expressed are solely those of the author(s) and do not necessarily reflect the position of European Publishing. European Publishing accepts no responsibility or liability for any consequences arising from the use of, or reliance on, this content.
